# IL-17A-associated IKK-α signaling induced TSLP production in epithelial cells of COPD patients

**DOI:** 10.1038/s12276-018-0158-2

**Published:** 2018-10-05

**Authors:** Giulia Anzalone, Giusy Daniela Albano, Angela Marina Montalbano, Loredana Riccobono, Anna Bonanno, Rosalia Gagliardo, Fabio Bucchieri, Roberto Marchese, Monica Moscato, Mirella Profita

**Affiliations:** 1 Institute of Biomedicine and Molecular Immunology “A. Monroy” (IBIM), National Research Council of Italy (CNR), Palermo, Italy; 2 Dipartimento di Biomedicina sperimentale e Neuroscienze Cliniche (BioNec), University of Palermo, Palermo, Italy; 3 Interventional Pulmonology Unit, La Maddalena Cancer Center, Palermo, Italy

## Abstract

Thymic stromal lymphopoietin (TSLP) is a cytokine expressed in the epithelium, involved in the pathogenesis of chronic disease. IL-17A regulates airway inflammation, oxidative stress, and reduction of steroid sensitivity in chronic obstructive pulmonary disease (COPD). TSLP and IL-17A were measured in induced sputum supernatants (ISs) from healthy controls (HC), healthy smokers (HS), and COPD patients by enzyme-linked immunosorbent assay. Human bronchial epithelial cell line (16HBE) and normal bronchial epithelial cells were stimulated with rhIL-17A or ISs from COPD patients to evaluate TSLP protein and mRNA expression. The effects of the depletion of IL-17A in ISs, an anticholinergic drug, and the silencing of inhibitor kappa kinase alpha (IKKα) on TSLP production were evaluated in 16HBE cells. Coimmunoprecipitation of acetyl-histone H3(Lys14)/IKKα was evaluated in 16HBE cells treated with rhIL-17A and in the presence of the drug. TSLP and IL-17A levels were higher in ISs from COPD patients and HS compared with HC. TSLP protein and mRNA increased in 16HBE cells and in normal bronchial epithelial cells stimulated with ISs from COPD patients compared with ISs from HC and untreated cells. IKKα silencing reduced TSLP production in 16HBE cells stimulated with rhIL-17A and ISs from COPD patients. RhIL-17A increased the IKKα/acetyl-histone H3 immunoprecipitation in 16HBE cells. The anticholinergic drug affects TSLP protein and mRNA levels in bronchial epithelial cells treated with rhIL-17A or with ISs from COPD patients, and IKKα mediated acetyl-histone H3(Lys14). IL-17A/IKKα signaling induced the mechanism of chromatin remodeling associated with acetyl-histone H3(Lys14) and TSLP production in bronchial epithelial cells. Anticholinergic drugs might target TSLP derived from epithelial cells during the treatment of COPD.

## Lung disease: inflammatory proteins implicated in chronic obstructive pulmonary disease

Two proteins involved in the maturation of immune cells contribute to the inflammation responsible for chronic obstructive pulmonary disease (COPD) and could thus provide promising drug targets for future therapies. Mirella Profita from the Institute of Biomedicine and Molecular Immunology “Alberto Monroy” in Palermo, Italy, and colleagues showed that lung secretions from people with COPD and those who smoke cigarettes have higher levels of interleukin-17A and thymic stromal lymphopoietin (TSLP), two proteins associated with inflammatory immune responses, than samples from healthy non-smokers. In bronchial epithelial cell lines, the researchers showed that interleukin-17A induces the expression of TSLP, whereas an existing bronchodilatator anticholinerc drug, usually used to treat COPD, reduces TSLP levels. The authors suggest that these drugs could complement existing COPD therapies to block proinflammatory proteins.

## Introduction

Chronic obstructive pulmonary disease (COPD) is characterized by airway inflammation and by a progressive airflow limitation usually caused by tobacco smoke^1^. The inflammation in COPD subjects is often resistant to corticosteroid treatments, and currently, there are no safe and effective alternative anti-inflammatory treatments^2^. The regular use of β2 adrenergic agonists and anticholinergic bronchodilators is recommended to maximize bronchodilation according to the current guidelines for the treatment of COPD^3,4^. Several studies provide perspectives on the use of muscarinic receptor antagonists for asthma and COPD, as these drugs acutely affect cholinergic airways obstruction and may have important beneficial effects on β_2_-agonist responsiveness, airway inflammation, and remodeling^5^. Many studies have proposed novel pharmacological strategies, including the use of anticholinergic drugs (Tiotropium) as anti-inflammatory and anti-remodeling drugs in COPD^5–7^.

Cigarette smoke-induced oxidative stress and nuclear factor kappa B (NFκB) activation decrease the anti-inflammatory effects of corticosteroids in the airways of COPD subjects^8,9^. NFκB regulates the production and activity of cytokines and chemokines associated with airway inflammation^10^. It is activated by phosphorylation, and the degradation of inhibitor kappa B (IκB) by IκB kinases (inhibitor kappa kinase alpha (IKKα) and IKKβ) leads to the nuclear translocation of NFκB and the transcription of NFκB-dependent genes^11^. IL-17A is a potent inducer of IL-8, a chemokine with a key role in the persistence of airway inflammation and in the reduction of steroid sensitivity, thereby exerting its action on human bronchial epithelial cells^12,13^.

Thymic stromal lymphopoietin (TSLP) is a cytokine of the IL-7 family produced mainly by stromal cells, including mast cells, and is involved in the activation, expansion, and survival of T lymphocytes and dendritic cells^14,15^. Its action is mediated by a heterodimeric receptor composed of IL-7Rα and TSLP receptor (TSLPR) in allergies and asthma^16^. The epithelial-derived TSLP is important for the initiation of allergic airway inflammation through a dendritic cell-mediated T helper 2 response. TSLP gene expression is controlled by inflammatory mediators, such as IL-1β and TNF-α, in a NFκB-dependent manner in airway epithelial cells^10^. Higher levels of TSLP are found in the bronchial mucosa of asthma and COPD patients, suggesting its involvement in the function and mechanisms of airway diseases as a signature of a “Th2-favoring”, besides as well as a “pro-allergic” cytokine^17^. An increased number of cells expressing TSLP mRNA are has been reported in the bronchi of patients with stable COPD and control smokers with normal lung function, suggesting additional roles for TSLP in COPD immune pathogenesis^18^. Airway structural cells produce and are targets of TSLP, suggesting a potential autocrine loop that may have a profound effect on the local inflammatory response and airway remodeling^17^. To our knowledge, no study has investigated the anti-inflammatory influence of anticholinergic drugs on the molecular mechanisms of IKKα activity in the control of IL-17A-mediated production of TSLP in bronchial epithelial cells.

We aimed to study the levels of TSLP and IL-17A present in the induced sputum supernatants (ISs) from COPD patients. Furthermore, we set up in vitro studies to investigate the potential role of rhIL-17A in chromatin remodeling and IKK-driven NFκB activation of TSLP gene transcription in bronchial epithelial cells during COPD pathogenesis. Finally, we analyzed the “in vitro” anti-inflammatory effects of anticholinergic drugs (generally used in the treatment of COPD as bronchodilators) on IL-17A-mediated TSLP production in bronchial epithelial cells.

## Materials and methods

### Patients

We recruited three groups of subjects: healthy asymptomatic non-smoking subjects with normal lung function (healthy controls; HC) (*n* = 10), symptomatic smokers with normal lung function (healthy smokers; HS) (*n* = 10), and smokers with COPD (*n* = 12). The diagnosis and severity assessment of COPD were defined and classified according to the criteria reported in the Global Initiative for Obstructive Lung Disease (GOLD) guidelines for COPD management (GOLD stage ≥ I)^19^. COPD subjects who experienced Exacerbations within 1 month of the study were excluded. Patients with COPD had a smoking history of 10 pack years or more.

All COPD patients were in a stable condition. COPD patients who had routine chest X-rays and computed tomographic scans showing obvious emphysema were excluded. All patients were characterized in terms of gender, age, smoking history, COPD symptoms, comorbidity, and treatment history. The exclusion criteria included the following: other systemic diseases, chronic bronchitis, chronic spontaneous sputum production, other lung diseases, upper and lower respiratory tract infections, treatment with glucocorticoids or anticholinergics within 3 months of the study and treatment with long-acting β_2_ adrenergic agonists in the 15 days preceding the study.

The bronchodilator **r**eversibility test was performed to exclude an asthmatic component. The increase in forced expiratory volume in the 1st second after salbutamol was lower than 12% and 200 ml compared with basal values in all COPD subjects.

The local Ethics Committee approved the study, and informed consent was obtained from each participating subject.

### Sputum induction and processing

Sputum induction and processing were performed according to the plug method. In brief, after the collection of the sputum, the selected plugs were processed with 4 × w/v of 0.1% dithiothreitol (DTT), with the subsequent addition of 4 × w/v phosphate-buffered saline (PBS) (PBS 1 × ; Gibco). The resulting suspension was vortexed for 30 s and then centrifuged at 1000 g for 20 min. The ISs were then aspirated and frozen at − 80 °C in separate aliquots for the subsequent biochemical analyses. The cells obtained from IS were then cytocentrifuged (Cytospin 2; Shandon, Runcorn, UK) and stained with May–Grunwald–Giemsa. To obtain differential cell counts, two independent investigators examined the slides blindly, counting at least 400 cells per slide.

### Measurement of TSLP and IL-17A

The levels of TSLP and IL-17A were measured in ISs using commercially available enzyme-linked immunosorbent assay kits (R&D Systems. Inc, MN, USA). The sensitivities of the kits were 9.87 pg/ml and 15 pg/ml for TSLP and IL-17A, respectively.

### Epithelial cell cultures

The SV40 large T antigen-transformed human bronchial epithelial cell line (16HBE) cell line (16HBE) was used for these studies. 16HBE is a cell line that retains the differentiated morphology and functions of normal airway epithelial cells. The cells represent a clonal diploid (2*n* = 6) cell line isolated from human lung. 16HBE cells were cultured as adherent monolayers in Eagle’s minimum essential medium (MEM) supplemented with 10% heat-inactivated (56 °C, 30 min) fetal bovine serum (FBS), 1% MEM (non-essential amino acids, Euroclone), 2 mM l-glutamine and 250 μg/ml gentamicin at 37 °C in a humidified 5% CO_2_ atmosphere.

### Lung biopsy collection and processing

Bronchoscopy with endobronchial biopsy was performed on six subjects. The biopsies were obtained from normal tissue. The tissue specimens obtained from bronchoscopic biopsy were placed in a tube with sterile Eagle’s MEM supplemented with 10% heat-inactivated (56 °C, 30 min) FBS, 1% MEM (non-essential amino acids, EuroClone), 2 mM l-glutamine, 500 μg/mL gentamicin, and 50 mg/mL fungizone at 37 °C in a humidified 5% CO2 atmosphere overnight. The next day, the biopsy samples were cut into smaller sample sizes to facilitate the epithelial cell culture and then placed in bovine collagen I (Gibco, Waltham, MA USA) coated 60-mm tissue culture dishes containing bronchial epithelial growth medium (BEGM, Lonza, Wokingham, UK).

### Human primary bronchial epithelial cell culture

The primary cells derived from bronchial tissue were successfully used to generate normal human bronchial epithelial cell cultures (NHBECs) after 6–7 days. When the epithelial cells reached 80–90% confluence, they were dissociated using trypsin-ethylenediaminetetraacetic acid (EDTA) and passaged onto collagen-coated plastic tissue culture plates (Corning Inc. Wilkes-Barre, PA) to be stimulated. Cells at passage (p)1 or 2 were used for experimentation. Control experiments confirmed that there were no significant differences between the responses of the cells at passages p1 and p2.

### Bronchial epithelial cell stimulation

16HBE cells (180,000 cells/well) were plated in standard six-well culture plates in MEM 10% FBS and grown to confluence (70–80%). After 1 h in 1 ml of MEM 1% FBS, the 16HBE cells were stimulated with ISs (20%) from HC (*n* = 6), HS (*n* = 6), and COPD (*n* = 6) subjects or with recombinant human (rh) IL-17A (20 ng/ml) (*n* = 6). Furthermore, ISs from COPD patients with the IL-17 concentrations closest to the median of the values were selected to stimulate 16HBE. A total of 200 μl of ISs was incubated with a rabbit polyclonal anti-human IL-17 antibody (H-132) (Santa Cruz Biotechnology, CA, USA) for 1 h at 37 °C to neutralize the specific activity before the stimulation of 16HBE cells (*n* = 6). To determine the effects of anticholinergic bronchodilator compounds on IL-17A activity, tiotropium Spiriva® (100 nM) (Boehringer Ingelheim Pharma GmbH & Co.KG, Biberach, Germany) was added to 16HBE cells and NHBECs for 30 min before the stimulation with ISs from COPD (*n* = 6) or with rhIL-17A (20 ng/ml) (*n* = 6). 16HBE were stimulated for 24 h for western blot and for 4 h to test for TSLP mRNA using real-time Polymerase chain reaction (PCR), IKKα coimmunoprecipitation and ChiP assay. Similar to the 16HBE cells, the NHBECs were cultured with rhIL-17A and with ISs from COPD patients to evaluate the TSLP expression by western blot analyses and real-time PCR.

### Total protein extraction

16HBE cells were washed with cold PBS and lysed in a buffer containing 10 mmol/L Tris–HCl (pH 7.4), 50 mmol/L NaCl, 5 mmol/L EDTA, and 1% Nonidet P-40. The phosphatase inhibitors included 20 mmol/L β-glycerophosphate, 0.3 mmol/L Na_3_VO_4_, and 1 mmol/L benzamidine (ICN Biochemicals, Inc, Aurora, OH, USA), and the protease inhibitors consisted of the complete protease inhibitor cocktail (Roche). The protein content of the supernatants was analyzed using the bicinchoninic acid assay (Pierce, Rockford, IL, USA); 25–30 μg of the lysate was then denatured under reducing conditions by boiling for 3 min in 50 mM Tris–HCl (pH 6.8), 1% sodium dodecyl sulfate (SDS), 2% β-mercaptoethanol, and 0.01% bromophenol blue. The total protein extracts were analyzed by western blot analysis.

The proteins were separated by SDS–polyacrylamide gel electrophoresis (PAGE) and transferred by electrophoresis onto Immobilon-P membranes (Millipore, Bedford, MA, USA). After the transfer, the membranes were blocked overnight at room temperature in PBS containing 3% bovine serum albumin and 0.5% Tween 20 and then incubated for 1 h at room temperature with the primary Abs. After washing, the blots were incubated for 45 min with the appropriate horseradish peroxidase-conjugated secondary Ab, and the bound Ab was detected using the ECL chemiluminescence detection system (Amersham-Pharmacia, Biotech), according to the manufacturer’s instructions. The membranes were stripped and reprobed with Ab against the housekeeping protein β-actin to normalize the differences in protein loading. Autoradiographic films were scanned and analyzed by densitometry using the NIH Image/Gel Plotting analysis program (National Institutes of Health, Bethesda, MD, USA). The results were normalized and expressed as ratios of the band intensities of the proteins and β-actin.

### Western blot antibodies

The following antibodies were used: rabbit anti-human TSLP (ProSci, Ca, USA) diluted to a ratio of 1:100, rabbit polyclonal anti-human IKKα Ab, clone 14A23 (Millipore, Ca, USA) diluted to 1:100; mouse monoclonal anti-human Ac-His H3 (Lys14) Ab, clone 13HH3-1A5 (Millipore, Ca, USA) diluted to 1:100; and mouse monoclonal anti-β-actin Ab (Sigma, St. Louis, MO, USA) diluted to 1:20,000.

### Quantitative RT-PCR of TSLP

Total RNA was extracted from 16HBE cells using the TRIzol Reagent (Invitrogen) following the manufacturer’s instructions and reverse-transcribed into complementary DNA using M-MLV-RT and oligo (dT)_12–18_ primers (Invitrogen). Quantitative real-time PCR of the TSLP transcript was conducted using the StepOne Plus Real-time PCR System (Applied Biosystems, Foster City, CA, USA) with specific FAM-labeled probes and primers (prevalidated TaqMan Gene expression assay for TSLP Hs00263639m1, Assays on Demand, Applied Biosystems). TSLP gene expression was normalized to that of the endogenous glyceraldehyde-3-phosphate dehydrogenase (GAPDH) control gene. The relative quantitation of gene expression was carried out using the comparative C_T_ method (2^−ΔΔCt^) and plotted as fold-change compared with to untreated cells as the reference sample.

### Silencing of IKKα

To confirm the role of IKKα in the expression of TSLP, we investigated the effects of IKKα silencing in human bronchial epithelial cells using specific short interfering RNA (siRNA) transfection. 16HBE cells were plated on six-well tissue culture plates and grown in medium containing 10% FBS without antibiotics until 60–80% confluence. IKKα siRNA (10 μM; Santa Cruz Biotechnology, Inc.) was then added to 100 μl of the siRNA transfection medium, and the reaction was performed according to the manufacturer’s instructions until complete cell transfection was achieved (7 h at 37 °C). For optimal siRNA transfection efficiency, siRNA (10 μM; Santa Cruz Biotechnology, Inc.) containing a scrambled sequence, which did not lead to the specific degradation of any known cellular mRNA, was used to control for the non-specific effects. Finally, the cells were stimulated with rhIL-17A or ISs from COPD (20%) patients for 24 h, and the total and nuclear proteins were extracted. The silencing efficacy of IKKα RNA interference using this method has been described in detail in a previous paper^18^.

### Co-immunoprecipitation

16HBE cells were washed with cold PBS 1 × before being lysed in a mild protein lysis buffer (50 mM Tris–HCl, 150 mM NaCl, 10 mM EDTA, and 0.1% Nonidet P-40) containing protease and phosphatase inhibitors. The cell lysate was pre-cleaned with protein A agarose beads (Protein A/GPlus-Agarose, Santa Cruz Biotechnology, Santa Cruz, CA, USA) and subsequently incubated overnight with rabbit polyclonal anti-acetyl-histone H3(Lys14) Ab (Millipore, CA, USA) (1:100) (pull-down). Protein A agarose beads were added and incubated for 1 h at 4 °C. The immunoprecipitates (IPs) were washed and boiled in 2 × SDS sample buffer for 5 min and centrifuged, and cell lysates were separated on 10% SDS/PAGE gels in preparation for western blotting with rabbit anti-human IKKα Ab. Non-immunized IgG was applied as the pull-down control to confirm the binding specificity. The total protein without IPs was run simultaneously as the control.

### ChiP assay

Chromatin immunoprecipitation (ChiP) analysis was performed according to the manufacturer′s instructions using the EZ-ChiP kit (UpstateBiotechnology, Inc., Lake Placid, NY, USA) in 16HBE cells. In brief, 37% paraformaldehyde was added to the culture medium to immobilize the DNA–protein and protein–protein interactions. Cells were washed twice with ice-cold PBS, resuspended in SDS cell lysis buffer containing protease inhibitor cocktail II, and kept in ice for 15 min. Cell lysates were sonicated on ice until the cross-linked chromatin was sheared to yield DNA fragments between 200 and 1000 bp. Each immunoprecipitation sample was diluted 10 times with the ChIP dilution buffer containing protease inhibitor cocktail II. Protein G agarose (Santa Cruz Biotechnology, CA, USA) was added to each immunoprecipitated sample; 50% of the supernatants were incubated overnight at 4 °C with rabbit polyclonal anti-acetyl-histone H3 (Lys14**)** Ab, and the other 50% were used as negative (normal mouse IgG, Millipore) and positive (anti-RNA polymerase II) controls. After the immunoprecipitation of the antibody/antigen/DNA complexes, the samples were eluted and subsequently treated with NaCl 5 M at 65 °C for 5 h to reverse the cros**s**-linking of protein/DNA complexes and free DNA. DNA purification was performed using spin columns according to the manufacturer’s instructions. PCR was performed with specific primers for 35 cycles, and amplified DNA fragments were analyzed on a 2% agarose gel by electrophoresis. PCR was performed using primers spanning the histone H3 binding site of the human TSLP gene promoter: 5′-GAGGGTCCAGAGCAATACAC-3′ and primer: 5′TGGAAGGGATATCAGAGAGG-3′. Purified DNA immunoprecipitated for Polymerase II was then analyzed by PCR using specific control primers.

## Results

### Demographic characteristics of patients and differential cell counts of IS

The patient characteristics are summarized in Table 1. According to the differential cell counts of the IS samples, we observed an increase in the number of both macrophages and neutrophils in HS, whereas COPD subjects showed a higher increase in the number of neutrophils and fewer macrophages than HS and HC. A higher number of eosinophils was reported in COPD subjects than in HC, whereas lymphocyte numbers did not differ between the three study groups (Table 2).

**Table 1:** Demographic characteristics of patients

	HC (*n* = 10)	HS (*n* = 10)	COPD (*n* = 12)
Age, year	60 ± 7	61.5 ± 8	64 ± 9
Gender, male/female	6/4	6/4	7/5
FEV1, % predicted	102.6 ± 12.6	105.2 ± 10	60.7 ± 14.5
FVC, % predicted	105.2 ± 16.7	109.1 ± 15	70.6 ± 19.2
FEV1/FVC (%)	91.6 ± 3.4	89.3 ± 4.9	61.4 ± 7.0
Smoking, pack/year	0	60 ± 18.0	63 ± 18.0

Data are presented as the mean ± SD. *HC* healthy asymptomatic non-smoking subjects with normal lung function, *HS* asymptomatic smokers with normal lung function, *COPD* subjects with chronic obstructive pulmonary disease, *FEV1* forced expiratory volume in one second, *FVC* forced vital capacity

**Table 2:** Differential cell count of induced sputum samples

	HC (*n* = 10)	HS (*n* = 10)	COPD (*n* = 12)
Macrophages (%)	80 (63.8–82.4)	51.9 (47.1–68.1)	21.6 (10.4–50.2)
Neutrophils (%)	17.2 (8–22.1)	46.1 (30.2–48.2)	71.8 (38.2–82.5)
Lymphocytes (%)	0.2 (0–0.8)	0.7 (0.4–1.2)	1.4 (0–1.3)
Eosinophils (%)	0.2 (0–0.6)	0.9 (0–1.4)	0.8 (0.2–2.0)
Epithelial cells (%)	0.6 (0.3–2)	0.5 (0–0.8)	0.3 (0–1.5)

Results are expressed as the median (25th to 75th percentiles). *HC* healthy asymptomatic non-smoking subjects with normal lung function, *HS* asymptomatic smokers with normal lung function, *COPD* subjects with chronic obstructive pulmonary disease

### Levels of TSLP and IL-17A in ISs

**Fig. 1:**
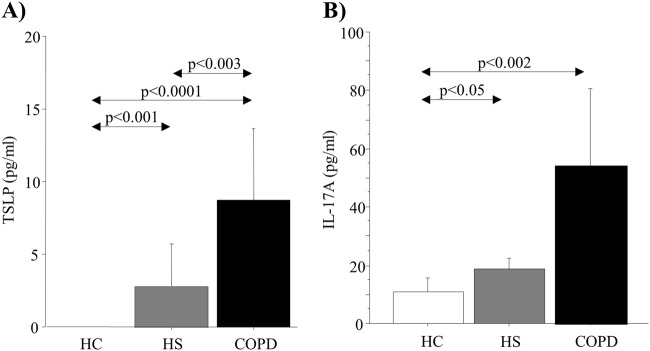
TSLP and IL-17A concentrations in ISs from HC (*n* = 10), HS (*n* = 10), and COPD patients (*n* = 12). **a** TSLP levels (pg/mL) and **b** IL-17A levels (pg/mL) were measured using specific commercially available kits as described in the “Materials and methods” section. Two technical replicates were performed. The bars represent the mean ± SD of the values (pg/mL). Statistical analysis was performed using the Kruskal–Wallis test followed by Bonferroni–Dunn correction for multiple comparisons. A *p* value < 0.05 was considered statistically significant

TSLP concentrations were significantly higher in ISs from HS and COPD patients compared with ISs from HC (*p* < 0.001 and *p* *<* 0.0001, respectively) and in ISs from COPD patients compared with ISs from HS (*p* < 0.003) (Fig. 1a). Furthermore, IL-17A concentrations were significantly higher in ISs from COPD and HS than in those from HC (*p* < 0.002 and *p* < 0.05, respectively) (Fig. 1b). Although the subjects used in this study are different, we obtained similar results to those previously observed^20^.

### TSLP production in 16HBE cells stimulated with rhIL-17A and ISs from COPD patients

**Fig. 2:**
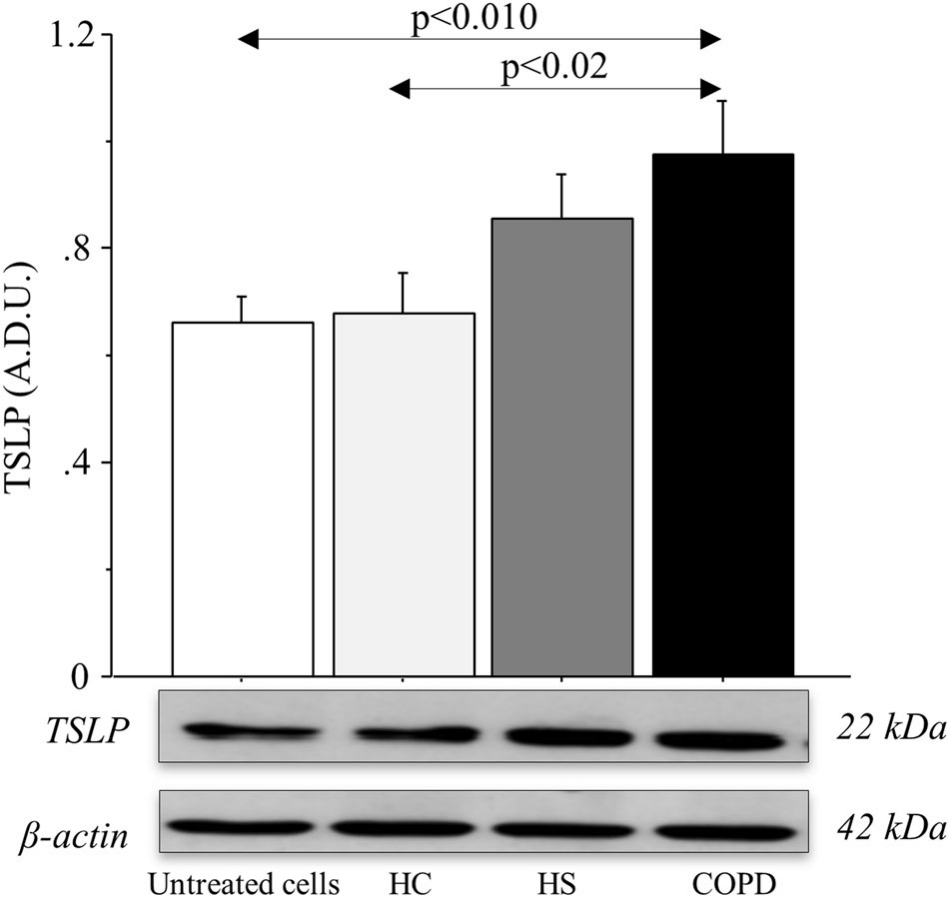
TSLP production in 16HBE cells stimulated with ISs from HC (*n* = 6), HS (*n* = 6), and COPD patients (*n* = 6). TSLP was detected in protein extracts by western blot analysis as described in the “Materials and methods” section. Bars represent the mean ± SD of arbitrary densitometric units (A.D.U.), normalized to β-actin, which was used as the loading control. Representative gel images of the experiments are shown. ANOVA with Fisher’s test correction was used to analyze data. A *p* value < 0.05 was considered statistically significant

**Fig. 3:**
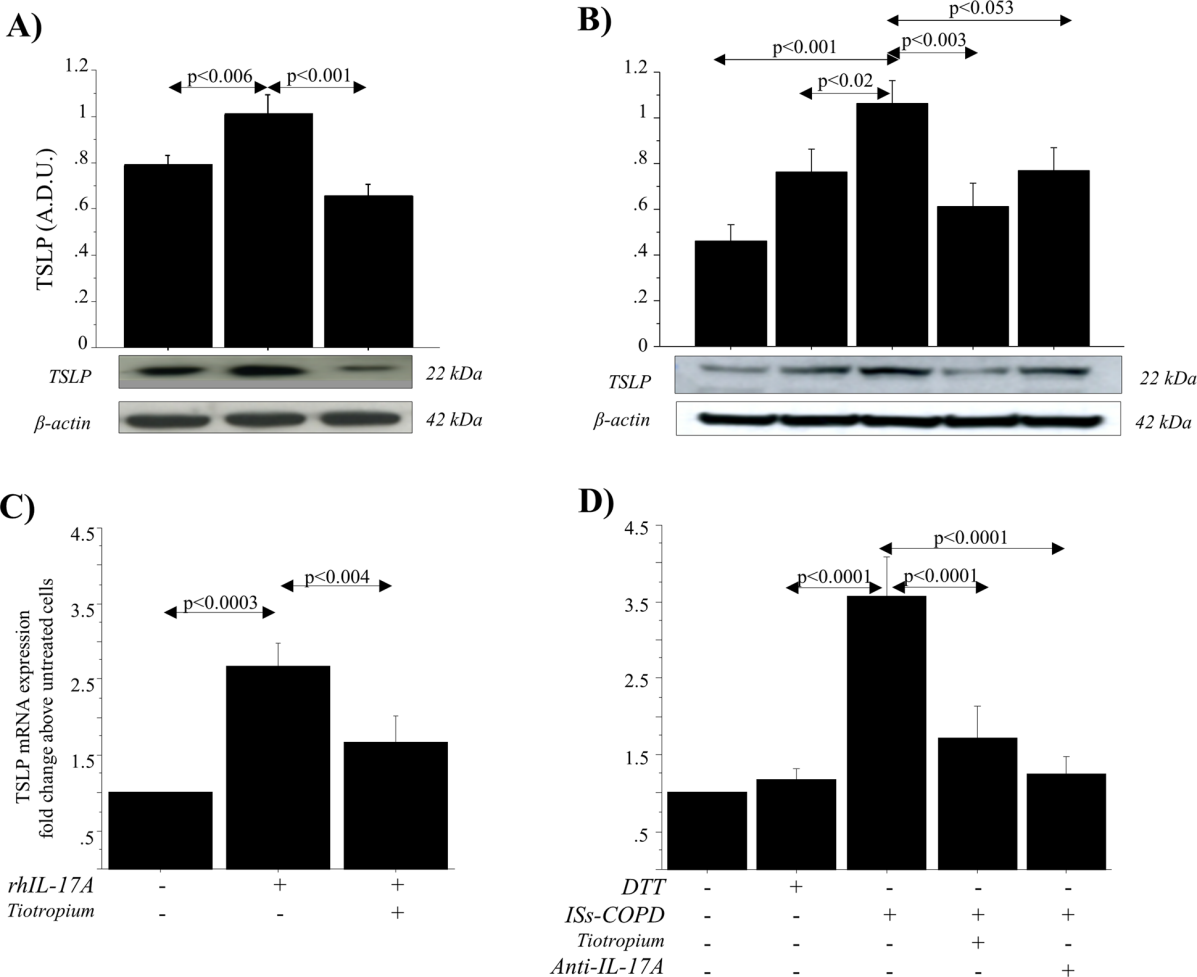
Effects of anticholinergic drugs on the production of TSLP in 16HBE cells stimulated with rhIL-17A or with ISs from COPD patients. 16HBE cells, in the presence or absence of Tiotropium (100 nM), were stimulated with rhIL-17A (20 ng/ml) or with ISs from COPD patients, untreated or treated with anti-IL-17A antibody, as well as with DTT (vehicle of ISs) (*n* = 6 for each experimental condition). **a** and **b** show TSLP protein detected by western blot analysis as described in the “Materials and methods” section. Representative gel images of the experiments are shown. Bars represent the mean ± SD of arbitrary densitometric units (A.D.U.), normalized to β-actin, which was used as the loading control. **c** and **d** show TSLP mRNA levels measured by real-time PCR as described in the Materials and Methods section (*n* = 3) and expressed as fold-change compared with untreated cells, which were chosen as the reference samples. ANOVA with Fisher’s test correction was used to analyze data. A *p* value < 0.05 was considered statistically significant

TSLP protein expression showed a significant increase in cell extracts from 16HBE cells stimulated with ISs from COPD patients compared with 16HBE cells stimulated with ISs from HC *(p* *<* 0.02) and untreated cells *(p* *<* 0.01) (Fig. 2). TSLP protein expression was significantly increased in cell extracts of 16HBE cells stimulated with rhIL-17A compared with untreated cells (*p* *<* 0.006). The treatment of the cells with tiotropium significantly reduced TSLP production in 16HBE cells stimulated with rhIL-17A (*p* *<* 0.001) (Fig. 3a). The analysis of TSLP production in protein extracts of 16HBE cells stimulated with ISs from COPD patients showed a significant increase in comparison to untreated cells or to cells treated with DTT (*p* *<* 0.001 and *p* *<* 0.02, respectively). The increased production of TSLP protein induced by ISs was significantly reduced when the cells were treated with tiotropium (*p* < 0.003) or with ISs from COPD patients treated with anti-IL-17A Ab to deplete the IL-17A present in the samples (*p* *<* 0.053) (Fig. 3b). RT-PCR analysis of TSLP showed higher levels of mRNA expression in 16HBE cells stimulated with 20 ng/ml rhIL-17A compared with the untreated cells or 16HBE cells treated with tiotropium (*p* *<* 0.0003 and *p* *<* 0.004, respectively) (Fig. 3c). Furthermore, 16HBE cells stimulated with ISs from COPD subjects showed a significant increase in TSLP mRNA expression (as indicated by the fewer amplification cycles required) compared with cells treated with DTT (*p* *<* 0.0001). 16HBE cells treated with tiotropium and 16HBE cells stimulated with ISs from COPD subjects treated with anti-IL-17A showed significantly lower levels of TSLP mRNA compared with cells stimulated with ISs from COPD patients (*p* *<* 0.0001 and *p* *<* 0.0001, respectively) (Fig. 3d). Treatment with anti-IL-17A Ab or tiotropium alone did not affect TSLP protein or mRNA production (data not shown) in 16HBE cells. Because tiotropium is currently used in the pharmacological treatment of these patients as a bronchodilator, we tested its potential anti-inflammatory activity only in samples containing ISs from COPD subjects.

### TSLP production in primary NHBE cells stimulated with rhIL-17A and ISs from COPD patients

**Fig. 4:**
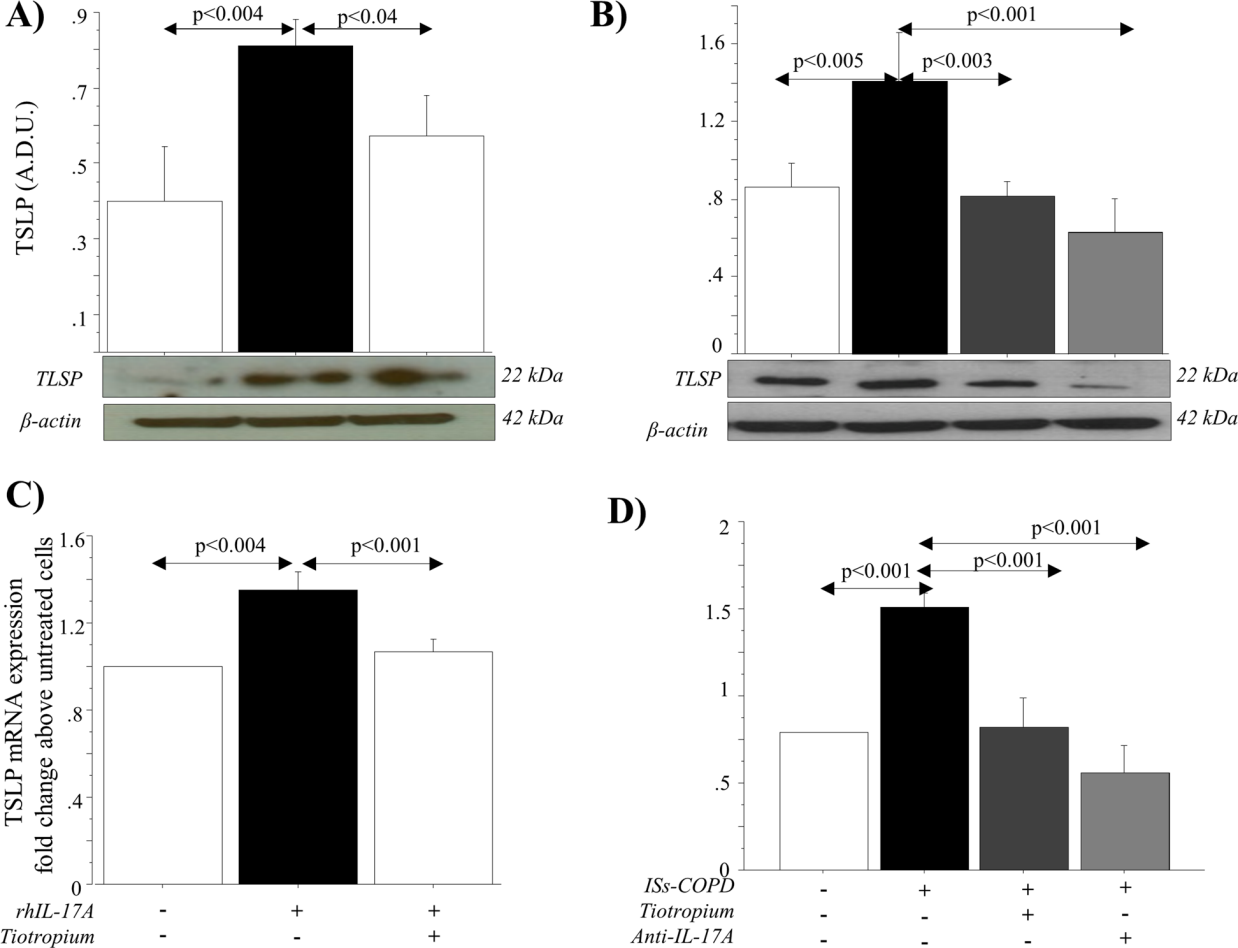
Effect of anticholinergic drugs on TSLP production in NHBE cells stimulated with rhIL-17A or with ISs from COPD patients. NHBE cells, in the presence or absence of Tiotropium (100 nM), were stimulated with rhIL-17A (20 ng/ml) (*n* = 3) or with ISs from COPD patients untreated or treated with anti-IL-17A antibody (*n* = 3) for each experimental condition). **a** and **b** show TSLP protein detected by western blot analysis as described in the “Materials and methods” section. Representative gel images of the experiments are shown. Bars represent the mean ± SD of arbitrary densitometric units (A.D.U.), normalized to β-actin used as the loading control. **c** and **d** show TSLP mRNA levels measured by real-time PCR as described in the “Materials and Methods” section and expressed as fold-change compared with untreated cells, chosen as the reference sample. ANOVA with Fisher’s test correction was used to analyze data. A *p* value < 0.05 was considered statistically significant

TSLP protein expression was statistically higher in NHBECs stimulated with rhIL-17A compared with untreated cells (*p* *<* 0.004) and decreased following the treatment with tiotropium (*p* *<* 0.04) (Fig. 4a). Furthermore, TSLP protein expression statistically increased when cells were stimulated with ISs from COPD patients compared with untreated cells (*p* *<* 0.005) and decreased following the treatment with tiotropium (*p* *<* 0.003) or with ISs from COPD treated with anti-IL-17A Ab (*p* *<* 0.001) (Fig. 4b). Real-time PCR analysis showed an increase in TSLP mRNA in NHBECs stimulated with ISs from COPD patients compared with untreated cells (*p* *<* 0.001). Real-time PCR analysis showed an increase in TSLP mRNA in NHBECs stimulated with rhIL-17A compared with untreated cells (*p* *<* 0.004). Pre-incubation of NHBECs with tiotropium significantly reduced the effects of rhIL-17A on TSLP protein production (*p* *<* 0.001) (Fig. 4c). Finally, the pre-incubation of NHBECs with tiotropium or the treatment of ISs with anti-IL-17A Ab significantly reduced the effects of ISs from COPD (*p* *<* 0.001 and *p* *<* 0.001, respectively) (Fig. 4d).

### Effect of IKKα silencing on TSLP expression

**Fig. 5:**
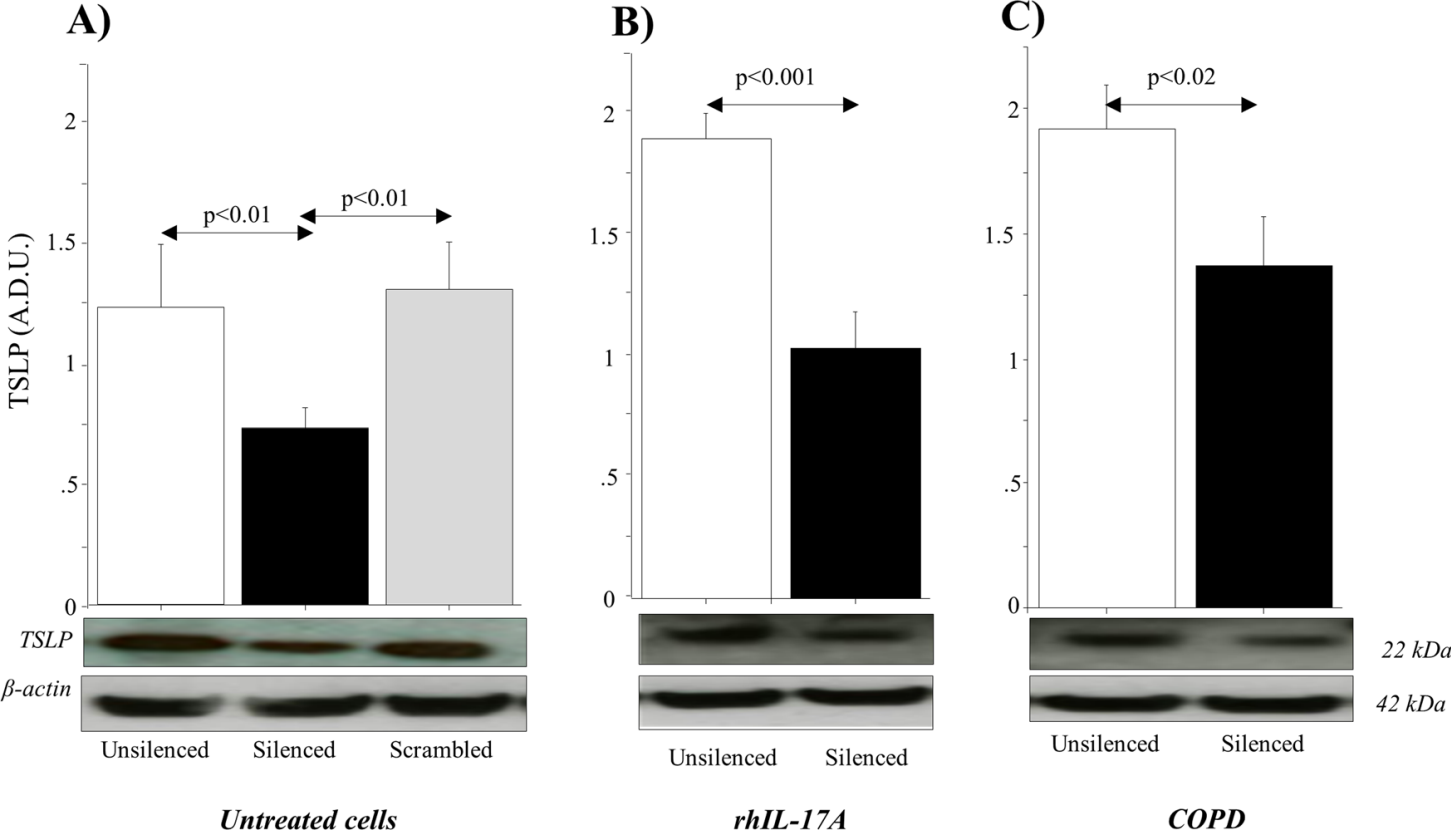
Effect of IKKα silencing on TSLP protein in 16HBE cells stimulated with rhIL-17A or ISs from COPD patients. **a** TSLP protein in unsilenced, silenced, and scrambled untreated cells (*n* = 3); **b** TSLP protein in unsilenced and silenced cells stimulated with rhIL-17A (*n* = 3); **c** TSLP protein in unsilenced and silenced cells stimulated with ISs from COPD (*n* = 3). TSLP protein was detected by western blot analysis as described in the “Materials and methods” section. Bars represent the mean ± SD of arbitrary densitometric units (A.D.U.), normalized to β-actin, which was used as the loading control. Representative gel images of the experiments are shown. ANOVA with Fisher’s test correction and the Student *t* test were used to analyze data. A *p* value < 0.05 was considered statistically significant

The silencing efficacy of our RNA interference method for IKKα signaling was 40% ± 3.6%, as previously described^20^. Temporary transfection of 16HBE cells with IKKα siRNA caused a statistically significant decrease of TSLP synthesis, as observed by western blot analysis. Particularly, the silencing of IKKα protein significantly decreased TSLP expression in untreated 16HBE cells (*p* *<* 0.01). Treating 16HBE cells with scrambled siRNA had no effect on the TSLP protein production compared with unsilenced cells (*p* *<* 0.01) (Fig. 5a). Finally, silencing the IKKα protein significantly decreased TSLP expression in cells stimulated with rhIL-17A (*p* *<* 0.001) (Fig. 5b) or in cells stimulated with ISs from COPD patients (*p* < 0.02) compared with unsilenced conditions (Fig. 5c).

### Co-immunoprecipitation of acetyl-histone H3/IKKα

**Fig. 6:**
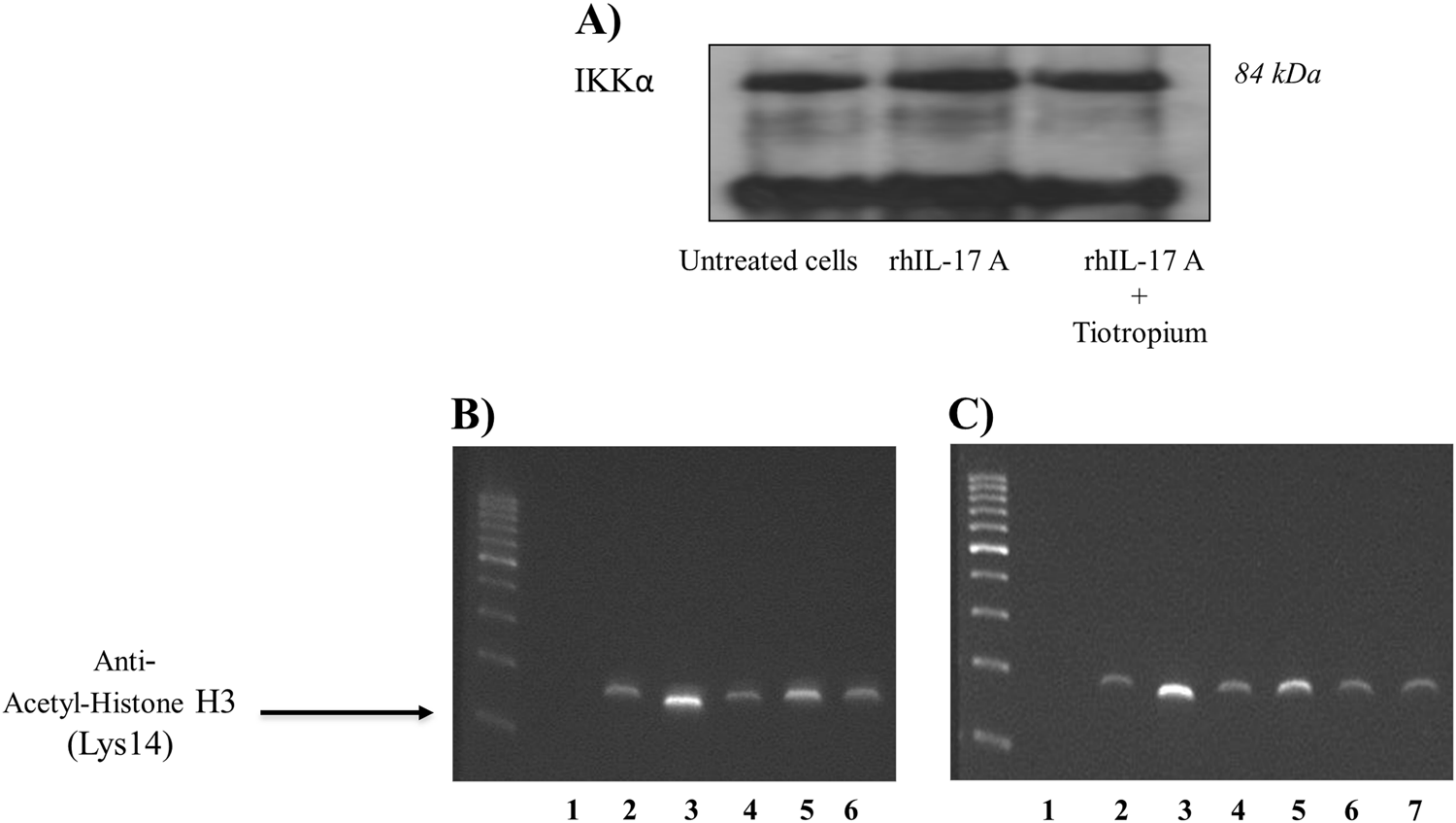
IKKα binding to acetyl-His H3 (Lys14) and acting on the promoter region of TSLP in epithelial cells. **a** Coimmunoprecipitation of IKKα and Ac-His H3 (Lys14) was performed in cell extracts from 16HBE cells stimulated for 4 h with rhIL-17A (20 ng/ml) in the presence or absence of tiotropium (*n* = 3). ChiP assay was performed using primers spanning the His H3 binding site of the human TSLP gene promoter: primer 1: 5′–3′; primer 2: 3′–5′; **b** the cells were stimulated with rhIL-17A (20 ng/ml), alone or in combination, for 4 h. Lane 1: negative control of PCR; lane 2: negative control of immunoprecipitation; lane 3: positive control of PCR; lane 4: untreated cells; lane 5: rhIL-17A (20 ng/ml); lane 6, rhIL-17A + tiotropium (*n* = 3). **c** the cells were stimulated with ISs alone or in combination for 4 h. Lane 1: negative control of PCR; lane 2: negative control of immunoprecipitation; lane 3: positive control of PCR; lane 4: untreated cells; lane 5: ISs from COPD patients; lane 6: ISs from COPD patients + tiotropium; line 7: ISs from COPD patients treated with anti-IL-17A Ab. Purified DNA was analyzed by PCR, using control primers specific for the GAPDH promoter. Representative gel images of the experiments are shown

The evaluation of the interactions between acetyl-histone H3 and IKKα proteins by coimmunoprecipitation showed that rhIL-17A (20 ng/ml) increased the cross-coupling between IKKα proteins and acetyl-histone H3 (Lys14). The treatment of cells with tiotropium significantly reduced the effect of rhIL-17A on the coimmunoprecipitation of IKKα proteins and acetyl-histone H3(Lys14) (Fig. 6a).

### Effects of IL-17A and ISs from COPD patients on chromatin

ChIP assays showed higher levels of anti-acetyl-histone H3(Lys14) Ab binding to the promoter region of TSLP in 16HBE cells stimulated with rhIL-17A (20 ng/ml) (Fig. 6b) or with ISs from COPD patients compared with untreated cells (Fig. 6b and c). Furthermore, we showed that the pretreatment of cells with tiotropium or ISs from COPD patients treated with the anti-IL-17A Ab generated a band with lower levels of intensity in the TSLP promoter region compared with cells stimulated with rhIL-17A or ISs alone.

## Discussion

**Fig. 7:**
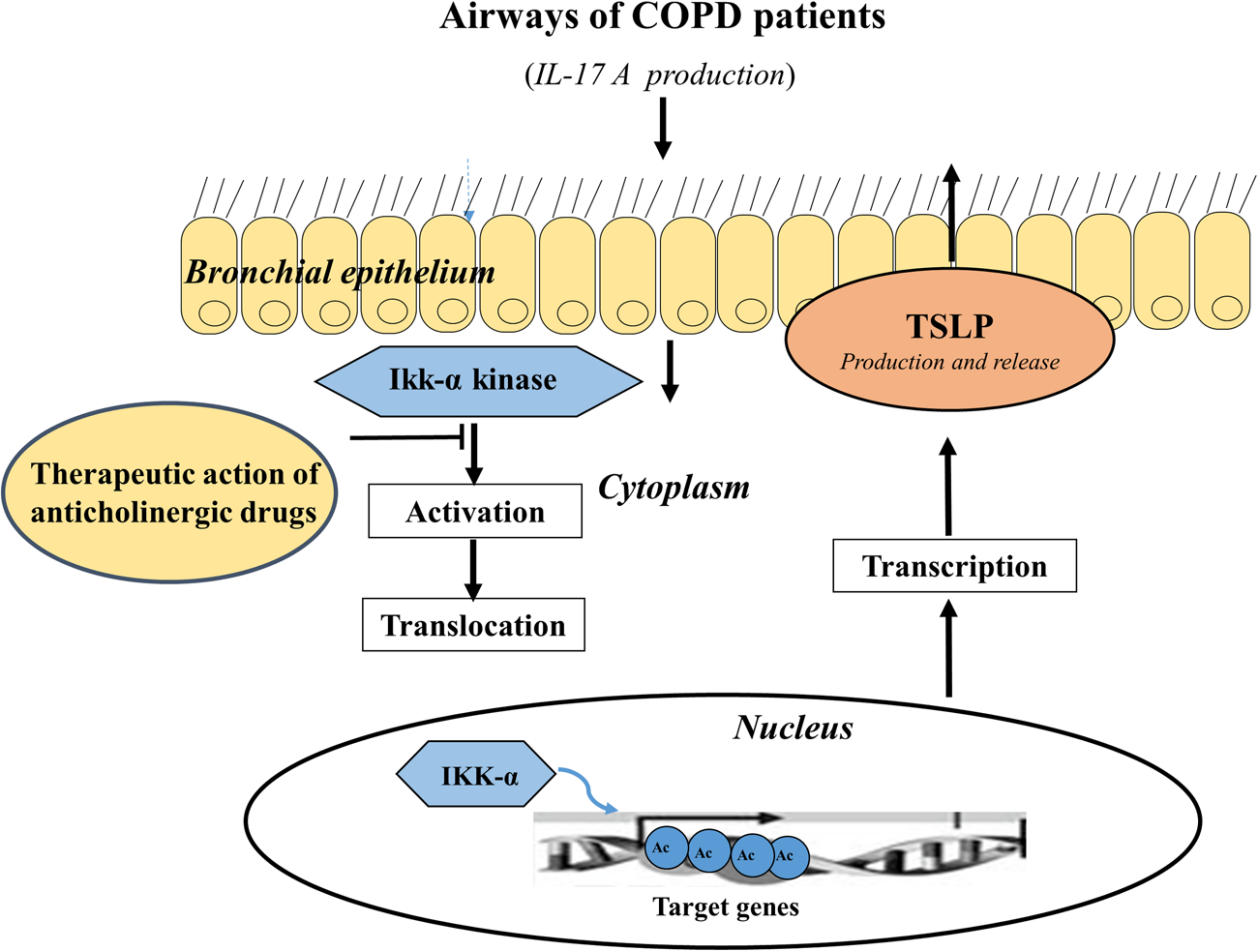
Graphical abstract. Summary of the study

In this study, we described the immunological link between IL-17A and TSLP levels observed in ISs from COPD patients. After studying the IL-17A and TSLP levels in COPD patients, we set up an “in vitro” model using 16HBE and NHBEC cells to illustrate how rhIL-17A or IL-17A found in the ISs from COPD subjects are involved in the production of TSLP in the airways. Furthermore, we described the molecular mechanisms of chromatin remodeling, associated with IL-17A-mediated IKKα activity and histone H3 acetylation in Lys14, which lead to an increase in TSLP mRNA transcription in bronchial epithelial cells. Finally, we provided new pharmacological perspectives on the anti-inflammatory role of anticholinergic drugs in the treatment of COPD patients and described their ability to attenuate IL-17A-mediated TSLP production (Fig. 7).

Epithelial cells are involved in the regulation of lung fluid balance, metabolism, clearance of inhaled agents, and secretion of mediators, most of which recruit and activate inflammatory cells in response to injuries in the airway^21,22^. Various invasive agents transduce the biological signals via an increase in the number of receptors in most adapted responses within the airway epithelium. Dysregulation of airway epithelial cell function contributes to the pathogenesis of major chronic inflammatory diseases of the lung, such as COPD, and the assessment of epithelial abnormalities includes phenotypic characteristics often associated with predicting a clinical benefit for epithelial-directed therapies^23^. TSLP is expressed predominantly by the epithelial cells of the lung^24,25^. TSLP mRNA and protein are elevated in asthma and COPD; however, its role in COPD is not yet well understood^26^. IL-17A has been found to be increased in submucosal biopsy specimens obtained from the large airways of COPD patients compared with control subjects^27^, and it is believed to be involved in epithelial cell activation^28^. On the other hand, Th17 immunity and the related cytokine IL-17A are involved in both innate and adaptive aspects of airway immunity, possibly representing a crucial cross-talk between the immune system and structural cells^29,30^. In our study, we detected higher levels of TSLP and IL-17A in the ISs from COPD patients and HS than in ISs from HC. These findings suggest a possible cross-talk between IL-17A found in the ISs and the activation of the bronchial epithelial cells that causes them to release TSLP during airway inflammation in COPD patients.

Differentiated cell cultures are an invaluable model for understanding the physiological properties of the human airway epithelium. Interactions between airway epithelium and environmental and inflammatory stimuli have been studied extensively in cell culture models of transformed 16HBE cell lines^13,28,30^. We studied the role of IL-17A in TSLP production in a 16HBE cell line stimulated with ISs from COPD, HS, and HC subjects, and our results show an increase in TSLP production in 16HBE cells stimulated with ISs from COPD subjects compared with cells treated with ISs from HC or untreated cells. Furthermore, we discovered that rhIL-17A increased TSLP production in 16HBE cells. We studied the effects of IL-17A depletion (anti-IL-17A Ab) in ISs from COPD on TSLP production in 16HBE cells and found a significant reduction of the production of TSLP mRNA transcripts and protein in 16HBE cells compared with cells stimulated with undepleted ISs. These findings highlight a direct and specific correlation between IL-17A and TSLP in the airways and show the involvement of IL-17A in the activation of bronchial epithelial cells that results in the release of TSLP during the inflammatory process of COPD. The validity and significance of these results are further supported by the data obtained from the experiments performed with normal bronchial epithelial.

A thorough knowledge of the molecular mechanisms of epigenetic changes in abnormal lung inflammation is important to fully understand the pathophysiology of COPD and may lead to the development of novel epigenetic therapies in the near future^31^. Chromatin modifications due to histone H3 acetylation in lysine (k9, K14, K27) lead to sustained proinflammatory gene transcription through different molecules. Such modifications facilitate the access of the transcription complex on the promoter gene^32^. IκB kinase (IKK)/NF-κB family signaling mediates the expression of hundreds of genes involved in inflammation, immune response, cell survival, and cancer^33,34^. The IKK complex contains two kinase subunits, IKKα and IKKβ. IκB kinase-driven nuclear factor-κB activation was observed in patients with asthma and COPD, and IKKα activity was higher in patients with COPD than in asthmatic patients^35^. Although IKKβ is predominantly cytoplasmic, IKKα has been found to shuttle between the cytoplasm and the nucleus and exert its functions as a chromatin kinase and acetylase, causing specific modifications of histones to generate expression of different genes in response to inflammatory stimuli^36^. Cigarette smoke-/TNFα as well as IL-17A induced the acetylation of histone H3 (K9) and inflammation via the differential activation of IKKα in human lung epithelial cells^20,37^. In our in vitro model of IL-17A mediated COPD inflammation, we found that IKKα silencing reduced TSLP synthesis in 16HBE cells stimulated with rhIL-17A or with ISs from COPD patients compared with the stimulated and unsilenced cells. These findings support the role of IKKα in the synthesis of TSLP through an IL-17A-mediated pathway in the airway epithelium of COPD patients. Finally, when the cellular extracts from 16HBE cells were co-immunoprecipitated with anti-acetyl-His H3 (Lys14) Ab and detected with anti-IKKα, higher levels of IKKα were found in stimulated 16HBE cells than in cells that were untreated or treated with an anticholinergic drug (Tiotropium). Additionally, a ChiP assay showed that the histone acetylated in Lys14 bound to the TSLP promoter region in cells stimulated with rhIL-17A or with ISs from COPD subjects. Depletion of IL-17A in ISs from COPD patients or epithelial cell treatment with tiotropium reduced this effect. These findings suggest that IL-17A found in the airways of COPD patients could potentially activate the synthesis of TSLP, inducing epigenetic chromatin remodeling in bronchial epithelial cells associated with IKKα acetylation of histone H3 at Lys14. We suggest that the anticholinergic drugs could represent a therapeutic approach that regulates the chromatin remodeling involved in the activation of inflammatory genes, such as TSLP, through IKKα acetylation of histone H3 at Lys14 in the airways of COPD patients. We previously reported that IL-17A in the airways of COPD patients induces chromatin remodeling and promotes the release of IL-8 in the bronchial epithelium and that anticholinergic drugs are able to control this proinflammatory activity^20^. For the present study, we used the same experimental ex vivo/in vitro model to study TSLP; however, we recruited a different number of HC subjects (10 rather than 14) and COPD patients (12 rather than 16) than for our previous study^20^. Furthermore, although the two studies appear to be similar, the current study showed that IL-17A-associated IKK-α signaling induced TSLP production by histone H3 acetylation at Lys14 in epithelial cells, rather than IL-8 production by histone H3 acetylation at Lys 9^20^. Finally, further studies are necessary to investigate the direct or indirect effects of IL-17A on IKKα signaling involved in IL-8 or TSLP production in bronchial epithelial cells.

The inhaled concentration of anticholinergic tiotropium is an 18-μg dose once-daily medication for COPD. It decreased rapidly in a multi-compartment manner as previously described^38,39^. Steady-state trough plasma concentrations were very low values (3–4 pg/mL). However, following treatment of the patients with a single 18-μg dose once per day, the overall concentration of tiotropium in the lungs is uncertain, and the physiologic concentration is unknown. For these reasons, we chose to use a tiotropium concentration of 100 nM, as it is commonly used in “in vitro” bronchial epithelial cell studies^20,39,40^.

In conclusion, our results contribute to a better understanding of the chromatin-mediated remodeling mechanisms associated with Th17 immunity and TSLP production in the airways of COPD patients, supporting the relevance of the role of chromatin modifications and deacetylases in COPD-induced lung inflammation^32^. Th17 immunity regulates the activation of TSLP production in bronchial epithelial cells, representing a key regulator of COPD pathogenesis. These data open up a new perspective on the importance of TSLP as a potential therapeutic target of anticholinergic drugs in COPD. Anticholinergic drugs showed a potential anti-inflammatory effect that might represent a useful innovative and alternative therapy to control IL-17A-induced production of mediators in epithelial cell derived from COPD patients. However, we recommend investigating the aforementioned mechanism in a larger population of COPD subjects and promoting adequate clinical trials to confirm the relevant contribution of anticholinergic drug therapy to controlling TSLP production in the airways.
